# circBTBD7 Promotes Immature Porcine Sertoli Cell Growth through Modulating miR-24-3p/*MAPK7* Axis to Inactivate p38 MAPK Signaling Pathway

**DOI:** 10.3390/ijms22179385

**Published:** 2021-08-30

**Authors:** Qiao Bian, Bin Chen, Bo Weng, Dan Chu, Xiangwei Tang, Saina Yan, Yanfei Yin, Maoliang Ran

**Affiliations:** College of Animal Science and Technology, Hunan Provincial Key Laboratory for Genetic Improvement of Domestic Animal, Hunan Agricultural University, Changsha 410128, China; bianqiao0808@163.com (Q.B.); wengbo831@126.com (B.W.); chudan0228@126.com (D.C.); Txw@stu.hunau.edu.cn (X.T.); yansaina99@126.com (S.Y.); lcyf9944@163.com (Y.Y.)

**Keywords:** circBTBD7, miR-24-3p, p38 MAPK, proliferation, porcine Sertoli cells

## Abstract

Sertoli cells are the crucial coordinators to guarantee normal spermatogenesis and male fertility. Although circular RNAs (circRNAs) exhibit developmental-stage-specific expression in porcine testicular tissues and have been thought of as potential regulatory molecules in spermatogenesis, their functions and mechanisms of action remain largely unknown, especially in domestic animals. A novel circBTBD7 was identified from immature porcine Sertoli cells using reverse transcription PCR, Sanger sequencing, and fluorescence in situ hybridization assays. Functional assays illustrated that circBTBD7 overexpression promoted cell cycle progression and cell proliferation, as well as inhibited cell apoptosis in immature porcine Sertoli cells. Mechanistically, circBTBD7 acted as a sponge for the miR-24-3p and further facilitated its target mitogen-activated protein kinase 7 (*MAPK7*) gene. Overexpression of miR-24-3p impeded cell proliferation and induced cell apoptosis, which further attenuated the effects of circBTBD7 overexpression. siRNA-induced *MAPK7* deficiency resulted in a similar effect to miR-24-3p overexpression, and further offset the effects of miR-24-3p inhibition. Both miR-24-3p overexpression and *MAPK7* knockdown upregulated the p38 phosphorylation activity. The SB202190 induced the inhibition of p38 MAPK pathway and caused an opposite effect to that of miR-24-3p overexpression and *MAPK7* knockdown. Collectively, circBTBD7 promotes immature porcine Sertoli cell growth through modulating the miR-24-3p/*MAPK7* axis to inactivate the p38 MAPK signaling pathway. This study expanded our knowledge of noncoding RNAs in porcine normal spermatogenesis through deciding the fate of Sertoli cells.

## 1. Introduction

Spermatogenesis is an extraordinarily complex and orchestrated process, which is mainly subdivided into three phases, including the mitosis, differentiation, and proliferation of spermatogonial stem cells, the meiotic of spermatocytes, and the differentiation of haploid spermatids [1]. In multiple spermatogenic cell types, Sertoli cells, the sole somatic cells within the seminiferous tubules, play key regulatory roles in guaranteeing the testis development, normal spermatogenesis process, and even male fertility [2,3]. In the neonatal and prepubertal periods, the immature and proliferation state, Sertoli cells arrest the meiosis progression of pro-spermatogonia, promote the Leydig cell differentiation, and further give priority to testis development and formation [2]. During puberty, Sertoli cells enter a mature and nonproliferation state, and their functional roles switch to guarantee the spermatozoa development through providing a stable microenvironment and morphogenetic support, as well as secreting several functional factors [3,4]. The proliferation of immature Sertoli cells determines the final number of Sertoli cells in adulthood, and further limits the daily sperm production, as each Sertoli cell has a fixed capacity to support a number of developing spermatozoa [5,6]. However, the regulatory mechanisms of Sertoli cell proliferation are poorly understood, especially in domestic animals.

Porcine Sertoli cells undergo two proliferation periods in the postnatal stages, including prepubertal and near puberty [7], and noncoding RNAs have been reported to be involved in porcine Sertoli cell proliferation. MicroRNAs (miRNAs), a class of small noncoding RNAs, are witnessed to participate in the regulation of proliferation and apoptosis of porcine Sertoli cells, including miR-762 [8], miR-638 [9], miR-1285 [10], miR-196a [11], miR-130a [12], and miR-499 [13]. Circular RNAs (circRNAs), a novel type of endogenous regulatory noncoding RNA, are characterized by the stable closed-loop structure and lack of 5′-3′ polarity [14]. It has been widely reported that circRNAs exhibit stage-dependent expression patterns in testis development of pig, sheep, and bovine [15,16,17,18], which indicates that circRNAs might play crucial regulatory roles in testis development and spermatogenesis. Existing literature has demonstrated that circRNAs participate in diverse biological processes through acting as an endogenous sponge of miRNA, including rolling circle translation, post-transcriptional regulation, circRNA-derived pseudogenes, and splicing interference [19,20]. However, circRNAs usually function as an endogenous sponge to modulate the activity and regulatory roles of miRNA in regulating cell proliferation, apoptosis, and differentiation [15,21]. Based on these abovementioned clues, we hypothesized that some specific circRNAs might also play crucial regulatory roles in the porcine Sertoli cell proliferation. 

In our previous studies, a large number of circRNAs are predicted from the RNA-seq data of sexually immature and mature porcine testicular tissues using a bioinformatics algorithm [16,22,23]. However, the functions and mechanisms of action of these identified circRNAs in porcine Sertoli cells remain largely unknown. In the present study, we found that circBTBD7, a novel circRNA originated from the BTB (POZ) domain containing 7 (*BTBD7*) gene, promotes cell cycle progression and cell proliferation, whereas it impedes cell apoptosis in immature porcine Sertoli cells. circBTBD7 elevates the mitogen-activated protein kinase 7 (*MAPK7*) gene expression through acting as an endogenous sponge of the miR-24-3p. MiR-24-3p results in the opposite effects through repression of *MAPK7* gene expression and inactivation of the p38 MAPK pathway, when compared with that of circBTBD7.

## 2. Results

### 2.1. Characteristics of circBTBD7 in Immature Porcine Sertoli Cells

**Figure 1:**
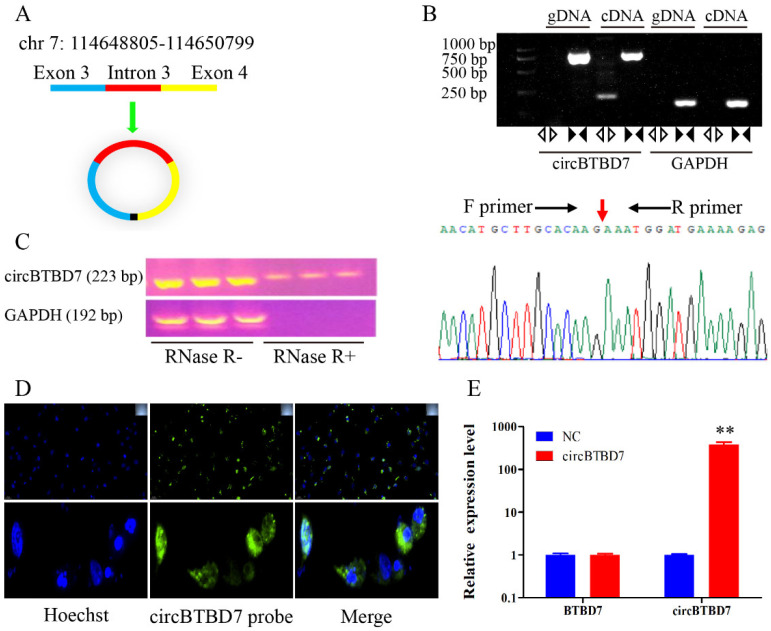
**Characteristics of circBTBD7 in immature porcine Sertoli cells.** (**A**) The sequence structure of circBTBD7 (Sus scrofa 11.1) predicted using a bioinformatics algorithm. (**B**) RT-PCR was conducted to validate the circBTBD7 sequence in cDNA and gDNA using divergent or convergent primers. The back-splice junction of circBTBD7 was further confirmed by Sanger sequencing. (**C**) The stability of circBTBD7 was validated by the RNase R digestion treatment. The total RNA from immature porcine Sertoli cells was treated with RNase R for 15 min before the RT-PCR assay. (**D**) The subcellular location of circBTBD7 was detected using the FISH assay. 100× (upper images) and 400× (bottom images). (**E**) The overexpression efficiency of circBTBD7 was measured using the qRT-PCR assay (*n* = 3). The *pig-TBP* gene was used as the internal control. Data are presented as the mean ± S.D. ** *p* < 0.01.

A novel circRNA, named circBTBD7, was predicted from our previous RNA-seq data using a bioinformatics algorithm, which derived from the exon 3 to 4 of *BTBD7* gene (1994 nt, *Sus scrofa* 11.1) (Figure 1A). Then, PCR analysis results demonstrated that the back-spliced junction of circBTBD7 was amplified with cDNA but not with genomic DNA using the divergent primers, whereas a specific fragment was amplified from both cDNA and genomic DNA using the convergent primers (Figure 1B). The back-spliced junction of circBTBD7 was further confirmed by Sanger sequencing (Figure 1B). Meanwhile, the circBTBD7 could resistant the RNase R digestion treatment compared to liner mRNA, for example, the *GAPDH* gene (Figure 1C). To analyze the primary mode of action of the circBTBD7, its subcellular location was checked in immature porcine Sertoli cells using RNA-FISH assay. The results showed that circBTBD7 was predominantly located in the cytoplasm rather than in the nucleus (Figure 1D). The qRT-PCR analysis result detected that circBTBD7 overexpression significantly elevated the circBTBD7 expression (*p <* 0.01), but had no effect on the *BTBD7* gene expression (Figure 1E). Overall, these results indicated that circBTBD7 was a stable circRNA in immature porcine Sertoli cells.

### 2.2. circBTBD7 Promotes Proliferation and Inhibits Apoptosis of Immature Porcine Sertoli Cells

**Figure 2:**
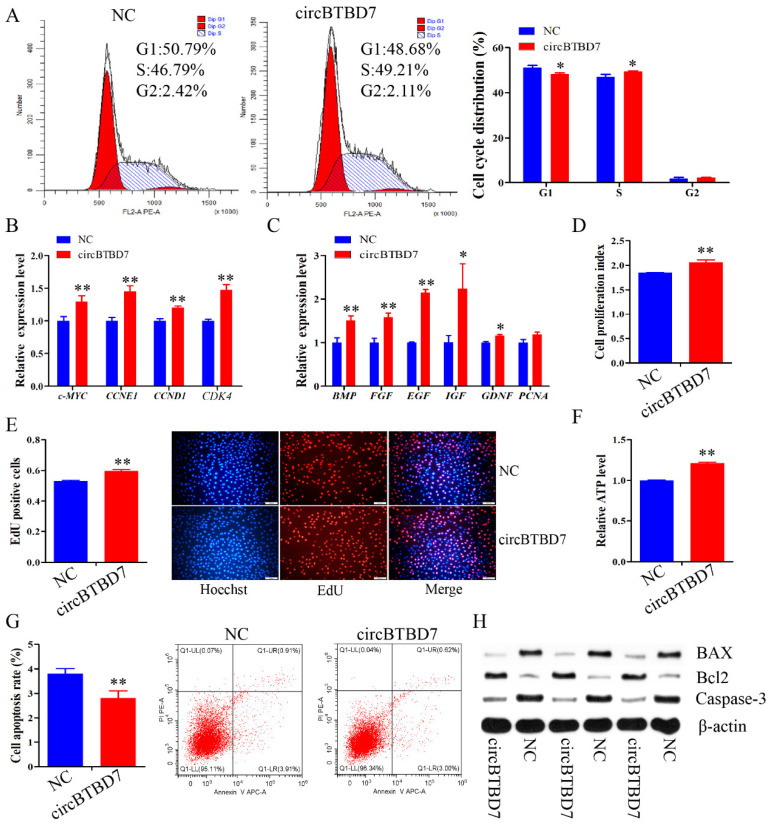
**circBTBD7 promotes proliferation and inhibits apoptosis of immature porcine Sertoli cells.** Immature porcine Sertoli cells were transfected with circBTBD7 overexpression vector or blank vector (NC). (**A**) The cell cycle distribution was analyzed using a FACSCanto II flow cytometer, and then the proportion of cells in G1, S, and G2 phases were counted (*n* = 3). (**B**,**C**) The mRNA expression of cell cycle-related genes (**B**) and cell proliferation marker genes (**C**) was measured using the qRT-PCR assay (*n* = 3). The *pig-TBP* gene was used as the internal control. (**D**) CCK-8 was used to explore the effect of circBTBD7 on the cell proportion index (*n* = 3). (**E**) The cell mitosis activity was checked using the EdU incorporation assay (*n* = 3). Representative images of EdU staining are listed. Scale bar = 200 μm. (**F**) The effect of circBTBD7 on the relative ATP level (*n* = 3). (**G**) The cell apoptosis rate was investigated using Annexin V-FITC/PI staining assay (*n* = 3). (**H**) The protein expression of cell survival-related geneswas determined using Western blot assay (*n* = 3). The β-actin gene was used as the internal control. Data are presented as the mean ± S.D. * *p* < 0.05, ** *p* < 0.01.

To investigate the physiological roles of circBTBD7, a gain-of-function assay was performed in immature porcine Sertoli cells. The cell cycle analysis results showed that circBTBD7 overexpression significantly decreased the percentage of cells in the G1 phase (*p* < 0.05), and significantly elevated that of in the S phase (*p* < 0.05) (Figure 2A). Meanwhile, the relative mRNA expression of cell cycle-related genes (*c-MYC*, *CCNE1*, *CCND1*, and *CDK4*) were significantly increased by circBTBD7 overexpression (*p* < 0.01) (Figure 2B). In addition, overexpression of circBTBD7 significantly upregulated the mRNA expression of cell proliferation-related genes, including *BMP*, *FGF*, *EGF*, *IGF*, and *GDNF* (*p* < 0.05) (Figure 2C). CCK-8 detection revealed that circBTBD7 overexpression significantly increased the cell proliferation index (*p* < 0.01) (Figure 2D). Similarly, EdU incorporation assay results demonstrated that the mitotic activity of cells was elevated by circBTBD7 overexpression (*p* < 0.01) (Figure 2E).

We further evaluated the effect of circBTBD7 on cell apoptosis. circBTBD7 overexpression induced a significantly higher relative ATP level compared with that of the NC group (*p* < 0.01) (Figure 2F). Additionally, the cell apoptosis rate was restrained by overexpression of circBTBD7 (*p* < 0.01) (Figure 2G). Furthermore, the protein expression of cell survival-related genes, *Bcl2*, *BAX*, and *Caspase-3*, were measured, and the results implied that circBTBD7 overexpression upregulated Bcl2 protein expression, and downregulated the protein expression of BAX and Caspase-3 (Figure 2G). Collectively, these results demonstrated that circBTBD7 promoted cell cycle progression and cell proliferation, and inhibited cell apoptosis in immature porcine Sertoli cells.

### 2.3. circBTBD7 Acts a Sponge for miR-24-3p

**Figure 3:**
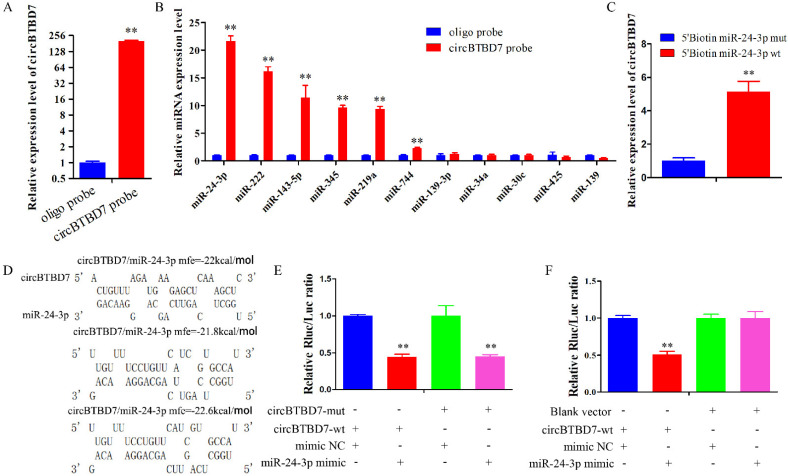
**circBTBD7 acts as a sponge of miR-24-3p.** (**A**) The efficiency of circBTBD7 probe used in RNA pull down assay was measured using the qRT-PCR assay (*n* = 3). (**B**) Effect of circBTBD7 on the abundance of eleven potential miRNAs (*n* = 3). The *U6* gene was used as the internal control for qRT-PCR assay. (**C**) Effect of miR-24-3p on the abundance of circBTBD7 (*n* = 3). (**D**) Predicted circBTBD7 binding sites to miR-24-3p. (**E**,**F**) Luciferase activity of four co-transfection cell groups in HEK293T cells (*n* = 3). Data are presented as the mean ± S.D. ** *p* < 0.01.

The RNA-FISH results indicated that circBTBD7 might play regulatory roles through acting as an miRNA sponge. To evaluate the potential mechanism of circBTBD7, eleven miRNAs were chosen from the overlap prediction results of TargetScan, RNAhybrid, and miRanda. Then, a specific biotin-probe of circBTBD7 was designed to conduct the RNA pull down assay in immature porcine Sertoli cells, and the effectiveness of pull down was significantly increased in cells with cirBTBD7 overexpression (*p* < 0.01) (Figure 3A). The qRT-PCR analysis results demonstrated that six miRNAs were significantly pulled down by the circBTBD7 (*p* < 0.01), including miR-24-3p, miR-222, miR-143-5p, miR-345, miR-219a, and miR-744 (Figure 3B). The miR-24-3p was chosen as a potential sponge target of circBTBD7 based on the highest enriched level (Figure 3B). Furthermore, circBTBD7 was also significantly pulled down by the miR-24-3p (*p* < 0.01) (Figure 3C). However, the result from dual-luciferase reporter assay showed that miR-24-3p induced a lower luciferase activity in both circBTBD7-wt and circBTBD7-mut transfected HEK293T cells (*p* < 0.01) (Figure 3D,E). Therefore, we further conducted another dual-luciferase reporter experiment using the blank vector, and the result demonstrated that miR-24-3p had no effect on the luciferase activity of blank vector transfected cells, whereas it reduced that of circBTBD7-wt transfected cells (*p* < 0.01) (Figure 3F). These data indicated that circBTBD7 acted as a sponge for the miR-24-3p.

### 2.4. miR-24-3p Suppresses Proliferation, Elevates Apoptosis of Immature Porcine Sertoli Cells

**Figure 4:**
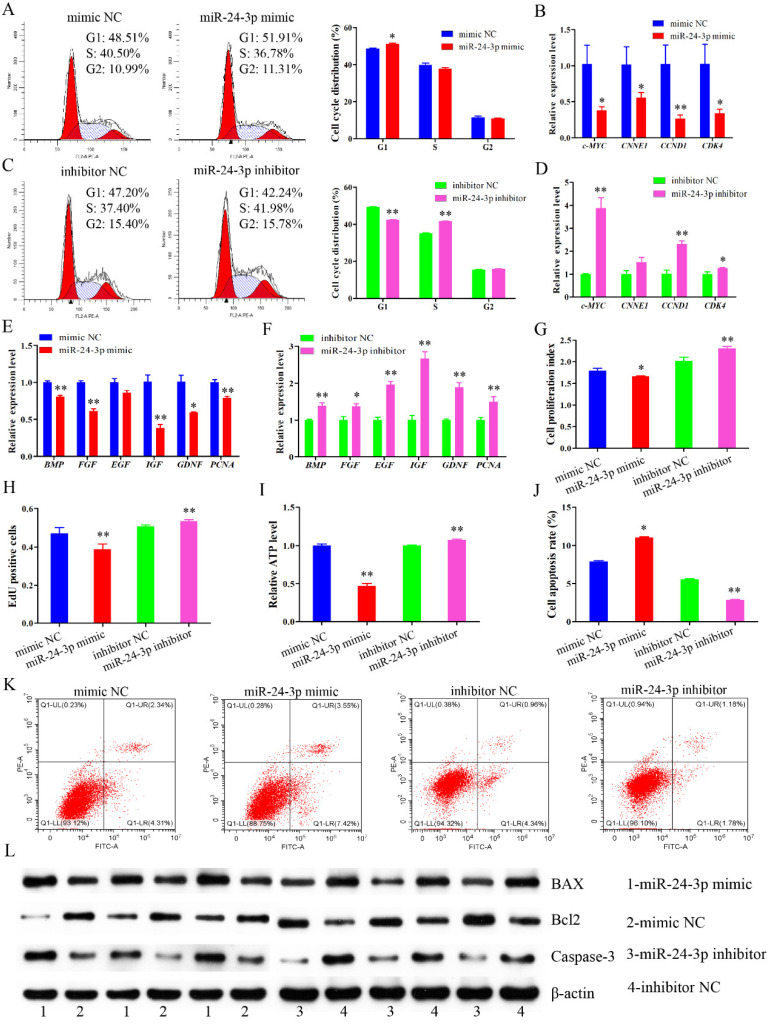
**miR-24-3p impeded proliferation and induces apoptosis of immature porcine Sertoli cells.** Immature porcine Sertoli cells were transfected with miR-24-3p mimic, mimic NC, miR-24-3p inhibitor, or inhibitor NC. (**A**,**C**) The cell cycle analysis of cells with miR-24-3p overexpression (**A**) or inhibition (**C**) (*n* = 3). (**B**,**D**) qRT-PCR assay measured the effect of miR-24-3p overexpression (**B**) or inhibition (**D**) on the mRNA expression of cell cycle-related genes (*n* = 3). (**E**,**F**) The mRNA expression of cell proliferation marker genes was illustrated in miR-24-3p mimic (**E**) or inhibitor (**F**) transfected cells (*n* = 3). (**G**,**H**) The effect of miR-24-3p overexpression or inhibition on cell proliferation was checked using the CCK-8 (**G**) and EdU (**H**) incorporation assays (*n* = 3). (**I**) The relative ATP level was investigated in 24-3p mimic or inhibitor transfected cells (*n* = 3). (**J**,**K**) Cell apoptosis rate was determined by Annexin V-FITC/PI staining followed by flow cytometry (*n* = 3). The cell apoptosis rate was counted in each cell transfection group, and representative images are listed. (**L**) The protein expression of cell survival-related genes was determined in miR-24-3p mimic or inhibitor transfected cells using Western blot assay (*n* = 3). Data are presented as the mean ± S.D. * *p* < 0.05, ** *p* < 0.01.

To explore the effects of miR-24-3p on cell proliferation and apoptosis, immature porcine Sertoli cells were transfected with an miR-24-3p mimic or inhibitor. The cell cycle analysis illustrated that miR-24-3p overexpression significantly elevated the G1 phase cell population (*p* < 0.05) (Figure 4A). Conversely, knockdown of miR-24-3p significantly decreased the percentage of cells in the G1 phase (*p* < 0.01), and upregulated that of those in the S phase (*p* < 0.01) (Figure 4C). Additionally, the relative mRNA expression of cell cycle-related genes was repressed by miR-24-3p overexpression (*p* < 0.05) (Figure 4B), whereas their expressions were increased by miR-24-3p knockdown (*p* < 0.05) (Figure 4D). Furthermore, miR-24-3p overexpression significantly downregulated the relative mRNA expression of cell proliferation-related genes (*p* < 0.05) (Figure 4E), whereas miR-24-3p knockdown significantly elevated their expressions (*p* < 0.05) (Figure 4F). The result from CCK-8 and EdU incorporation assays both showed that the cell mitotic activity was significantly decreased by miR-24-3p overexpression (*p* < 0.01), and increased by miR-24-3p knockdown (*p* < 0.01) (Figure 4G,H).

Next, we further measured the effect of miR-24-3p on cell apoptosis. miR-24-3p overexpression resulted in a decrease of the relative ATP level (*p* < 0.01), whereas miR-24-3p knockdown induced a higher relative ATP level in immature porcine Sertoli cells (*p* < 0.01) (Figure 4I). Similarly, the cell apoptosis rate was significantly elevated in miR-24-3p mimic transfected cells (*p* < 0.05), whereas it was significantly decreased in miR-24-3p knockdown cell group (*p* < 0.01) (Figure 4J,K). We further measured the protein expression of Bcl2, BAX, and Caspase-3, and detected that miR-24-3p overexpression downregulated Bcl2 expression and elevated the expression of BAX and Caspase-3, whereas knockdown of miR-24-3p caused an opposite result (Figure 4L). Taken together, these results indicated that miR-24-3p repressed cell cycle progression and cell proliferation, as well as further induced cell apoptosis in immature porcine Sertoli cells.

### 2.5. miR-24-3p Antagonizes the Effects of circBTBD7 on Immature Porcine Sertoli Cells

**Figure 5:**
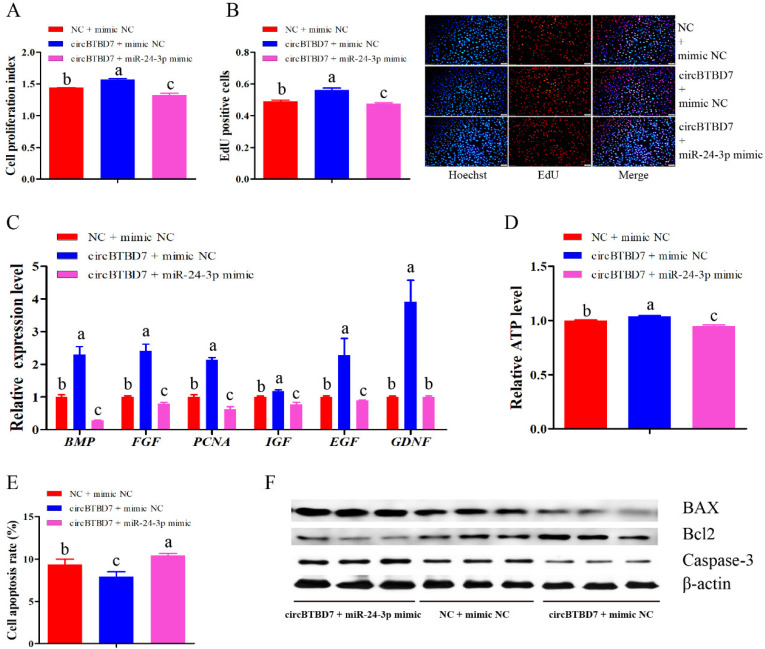
**miR-24-3p attenuates the effects of circBTBD7 in immature porcine Sertoli cells.** Three co-transfection treatments were performed in this section, including NC + mimic NC, cirBTBD7 + mimic NC, circBTBD7 + miR-24-3p mimic. The cell proliferation of each co-transfection group was measured using CCK-8 (**A**) and EdU (**B**) incorporation assays (*n* = 3). The representative images of EdU incorporation assay are listed. Scale bar = 200 μm. (**C**) The mRNA expression of cell proliferation marker genes was checked using the qRT-PCR assay (*n* = 3). The *pig-TBP* gene was used as the internal control. (**D**) The relative ATP level was measured in each co-transfection group (*n* = 3). (**E**) The cell apoptosis rate was detected and counted using the Annexin V-FITC/PI staining assay (*n* = 3). (**F**) Western blot assay was used to detect the protein expression of cell survival-related genes in each co-transfection group (*n* = 3). Data are presented as the mean ± S.D. Different letters mean values within each section were significantly different.

To further confirm that miR-24-3p participated in the regulatory roles of circBTBD7 on immature porcine Sertoli cells, three co-transfection treatments were conducted, including the vector NC + mimic NC, circBTBD7 + mimic NC, and circBTBD7 + miR-24-3p mimic. The CCK-8 and EdU incorporation results indicated that the cell proliferation activity was significantly elevated by the circBTBD7 + mimic NC treatment (*p* < 0.01), whereas it was reduced by the circBTBD7 + miR-24-3p mimic treatment when compared with that of the vector NC + mimic NC treatment (*p* < 0.01) (Figure 5A,B). Similarly, circBTBD7-induced higher relative mRNA expression of cell proliferation-related genes was restrained by miR-24-3p overexpression (*p* < 0.05) (Figure 5C). Correspondingly, the relative ATP level was also increased by the circBTBD7 overexpression (*p* < 0.05), whereas this effect was offset by the overexpression of miR-24-3p (*p* < 0.05) (Figure 5D). In addition, detection using the Annexin V-FITC/PI staining assay demonstrated that circBTBD7 induced a lower cell apoptosis rate (*p* < 0.05), which was significantly upregulated by the miR-24-3p overexpression (*p* < 0.05) (Figure 5E). Consistently, circBTBD7 elevated Bcl2 protein expression and downregulated the protein expression of BAX and Caspase-3, whereas these effects were attenuated by the miR-24-3p (Figure 5F). These abovementioned results indicated that circBTBD7 promoted proliferation and suppressed apoptosis in immature porcine Sertoli cells through sponging miR-24-3p. 

### 2.6. miR-24-3p Binding to the MAPK7 Gene and Controls Its Expression

**Figure 6:**
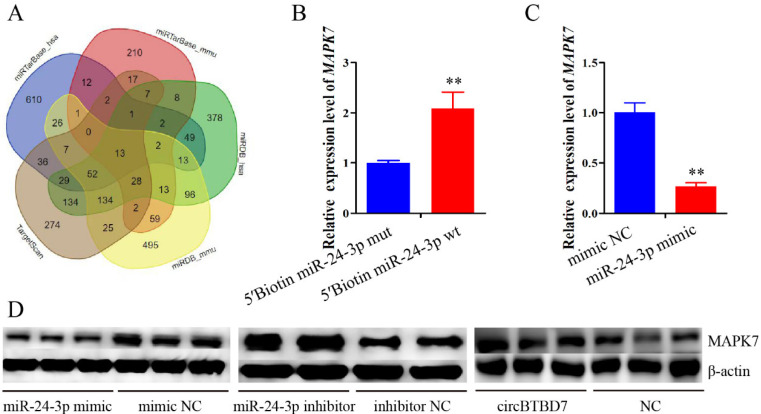
**miR-24-3p binding to the *MAPK7* gene and controls its expression.** (**A**) The potential target genes of miR-24-3p were predicted using miRTarBase, TargetScan, and miRDB online software. (**B**) RNA pull down and qRT-PCR assays were used to check the bind relationship between circBTBD7 and miR-24-3p. (**C**,**D**) The *MAPK7* mRNA and protein expression were detected using qRT-PCR (**C**) and Western blot (**D**) assays. The *pig-TBP* and *β-actin* genes were used as the internal control for qRT-PCR and Western blot assays, respectively. Data are presented as the mean ± S.D. ** *p* < 0.01.

To explore the mechanism of miR-24-3p in immature Sertoli cell proliferation and apoptosis, its target genes were predicted using the TargetScan, miRDB, and miRTarBase online software (Figure 6A). The *MAPK7*, a well-known element of the MAPK signaling pathway, was chosen as a potential target gene through overlapping the prediction results. Then, the results from RNA pull down and qRT-PCR assays indicated that the *MAPK7* was significantly pulled down by the miR-24-3p (*p* < 0.01) (Figure 6B). Additionally, the mRNA and protein expression of the *MAPK7* gene were both significantly decreased by the overexpression of miR-24-3p (*p* < 0.01) (Figure 6C,D). However, the MAPK7 protein expression was upregulated by the circBTBD7 (Figure 6D). Collectively, the *MAPK7* gene was a directed target of miR-24-3p, and its protein expression was inhibited by the miR-24-3p but increased by circBTBD7.

### 2.7. MAPK7 Knockdown Attenuates the Effects of miR-24-3p Inhibition on Immature Porcine Sertoli Cells

**Figure 7:**
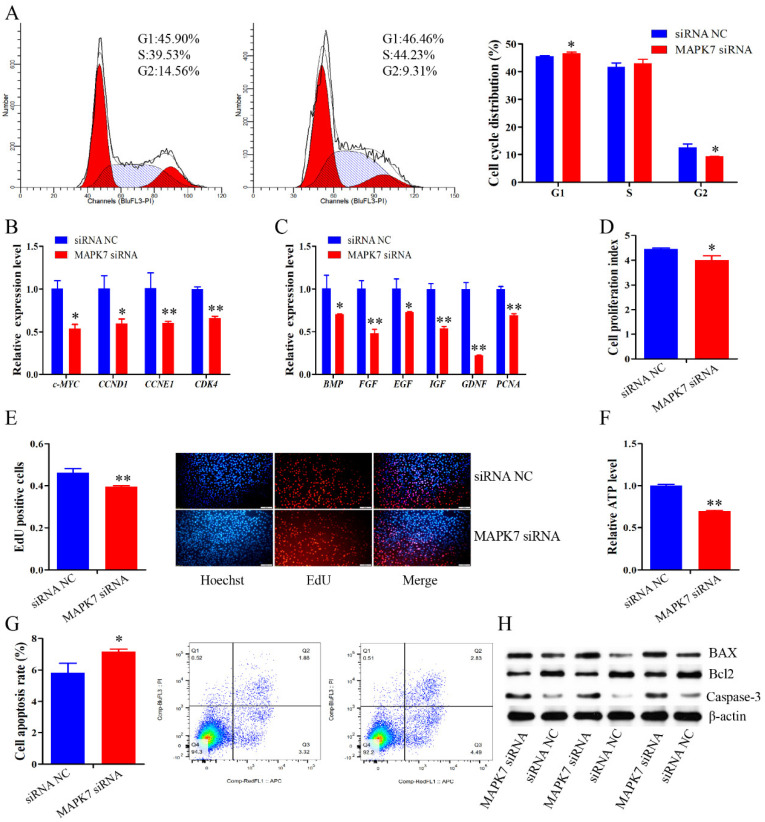
***MAPK7* gene deficiency inhibits immature Sertoli cell proliferation and promotes cell apoptosis.** A specific siRNA of *MAPK7* gene was transfected in immature porcine Sertoli cells to knockdown its expression. (**A**) The cell cycle distribution was analyzed and then the proportion of cells in G1, S, and G2 phases were counted (*n* = 3). (**B**,**C**) The mRNA expression of cell cycle-related genes (**B**) and cell proliferation marker genes (**C**) were measured using the qRT-PCR assay (*n* = 3). The *pig-TBP* gene was used as the internal control. (**D**,**E**) CCK-8 (**D**) and EdU incorporation (**E**) assays were used to explore the effect of *MAPK7* knockdown on the cell proportion (*n* = 3). Representative images of EdU staining are listed. Scale bar = 200 μm. (**F**) The effect of *MAPK7* knockdown on the relative ATP level (*n* = 3). (**G**) The cell apoptosis rate was investigated using Annexin V-FITC/PI staining assay (*n* = 3). (**H**) The protein expression of cell survival-related genes was determined using Western blot assay (*n* = 3). The β-actin gene was used as the internal control. Data are presented as the mean ± S.D. * *p* < 0.05, ** *p* < 0.01.

To clarify the biological effects of the *MAPK7* gene on cell proliferation and apoptosis, a specific siRNA was transfected in cells to knockdown its expression. Cell cycle analysis showed that *MAPK7* knockdown significantly increased the cell population in G1 phase (*p* < 0.05), and decreased the percentage of cells in G2 phase (*p* < 0.05) (Figure 7A). The qRT-PCR assay demonstrated that the relative mRNA expression of cell cycle and proliferation-related genes were both decreased by the inhibition of *MAPK7* (*p* < 0.05) (Figure 7B,C). Furthermore, CCK-8 and EdU incorporation assay results indicated that the mitotic activity was significantly decreased by siRNA-induced *MAPK7* inhibition (*p* < 0.05) (Figure 7D,E). In addition, the relative ATP level was significantly decreased (*p* < 0.01) (Figure 7F), whereas the cell apoptosis rate was significantly elevated when the cell was transfected with *MAPK7* siRNA (*p* < 0.05) (Figure 7G). Similarly, *MAPK7* gene deficiency inhibited the Bcl2 protein expression, and promoted the protein expression of BAX and caspase-3 (Figure 7H). These results indicated that *MAPK7* knockdown inhibited cell proliferation, and induced cell apoptosis in immature porcine Sertoli cells, which was similar with that of miR-24-3p overexpression.

**Figure 8:**
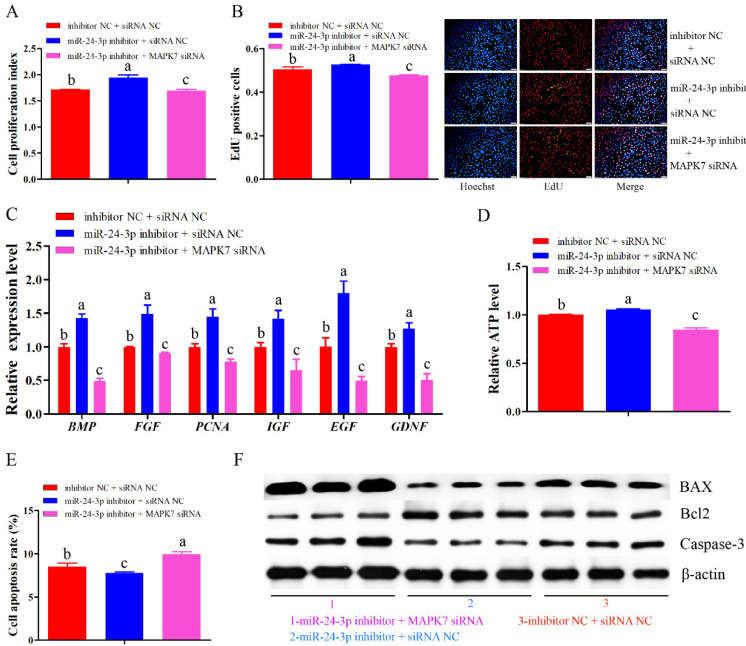
***MAPK7* knockdown abolishes the roles of miR-24-3p inhibition in immature porcine Sertoli cells.** Three co-transfection treatments were performed in this section, including inhibitor NC + siRNA NC, miR-24-3p inhibitor + siRNA NC, miR-24-3p inhibitor + MAPK7 siRNA. (**A**,**B**) The cell proliferation of each co-transfection group was measured using CCK-8 (**A**) and EdU (**B**) incorporation assays (*n* = 3). The representative images of EdU incorporation assay are listed. Scale bar = 200 μm. (**C**) The mRNA expression of cell proliferation marker genes was checked using the qRT-PCR assay (*n* = 3). The *pig-TBP* gene was used as the internal control. (**D**) The relative ATP level was measured in each co-transfection group (*n* = 3). (**E**) The cell apoptosis rate was detected and counted using the Annexin V-FITC/PI staining assay (*n* = 3). (**F**) Western blot assay was used to detect the protein expression of cell survival-related genes in each co-transfection group (*n* = 3). The β-actin gene was used as the internal control. Data are presented as the mean ± S.D. Different letters mean values within each section were significantly different.

Then, three co-transfection treatments were further conducted to further determine whether the *MAPK7* gene participated in mediating the regulatory mechanism of miR-24-3p, including inhibitor NC + siRNA NC, miR-24p-3p inhibitor + siRNA NC, and miR-24-3p inhibitor + MAPK7 siRNA. The results from CCK-8 and EdU incorporation assays indicated that miR-24-3p inhibition induced a higher proliferation activity of cells (*p* < 0.01), which was offset by the *MAPK7* knockdown (*p* < 0.05) (Figure 8A,B). Similarly, *MAPK7* knockdown also could restrain the miR-24-3p inhibition-induced higher relative mRNA expression of cell proliferation-related genes (*p* < 0.05) (Figure 8C). In addition, miR-24-3p inhibition caused a higher relative ATP level and a lower cell apoptosis rate in immature porcine Sertoli cells (*p* < 0.05), whereas this effect was attenuated by the knockdown of *MAPK7* (*p* < 0.05) (Figure 8D,E). Correspondingly, the effect of miR-24-3p inhibition on the protein expression of Bcl2, BAX, and Caspase-3 was also abolished by the *MAPK7* knockdown (Figure 8F). Overall, these data showed that *MAPK7* knockdown attenuated the effects of miR-24-3p inhibition on immature porcine Sertoli cells.

### 2.8. miR-24-3p Activates the p38 MAPK Signaling Pathway

**Figure 9:**
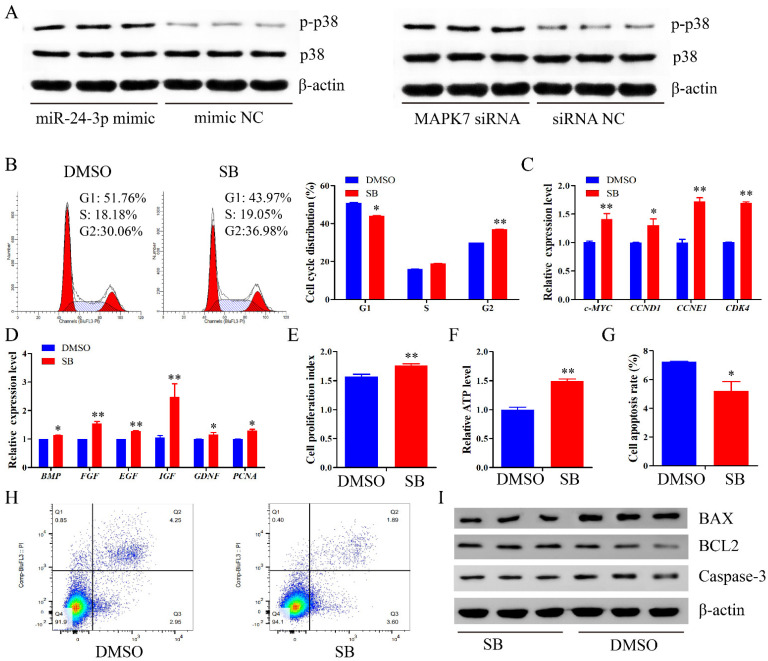
**miR-24-3p activates the p38 MAPK signaling pathway.** SB202190 (SB) was transfected in immature porcine Sertoli cells to induce the p38 MAPK signaling pathway inhibition. The SB was diluted by the DMSO regent. (**A**) Western blot assay was used to investigate the effect of miR-24-3p overexpression and *MAPK7* inhibition on the protein expression of p38 and p-p38. (**B**) The cell cycle distribution was analyzed and then the proportion of cells in G1, S, and G2 phases were counted (*n* = 3). (**C**,**D**) The mRNA expression of cell cycle-related genes (**C**) and cell proliferation marker genes (**D**) was measured using the qRT-PCR assay (*n* = 3). The *pig-TBP* gene was used as the internal control. (**E**,**F**) CCK-8 (**E**) and EdU incorporation (**F**) assays were used to explore the effect of *MAPK7* knockdown on the cell proportion (*n* = 3). Representative images of EdU staining are listed. (**G**). The relative ATP level was measured using an ATP assay kit (*n* = 3). (**H**) The cell apoptosis rate was investigated using Annexin V-FITC/PI staining assay (*n* = 3). (**I**) The protein expression of cell survival-related genes was determined using Western blot assay (*n* = 3). The β-actin gene was used as the internal control. Data are presented as the mean ± S.D. * *p* < 0.05, ** *p* < 0.01.

We then sought to explore whether the MAPK signaling pathway could meditate the regulatory roles of miR-24-3p. The Western blot assay demonstrated that both miR-24-3p overexpression and *MAPK7* knockdown elevated the p-p38 protein expression but had no obvious effect on the total p38 protein expression (Figure 9A). To measure the effects of p38 MAPK signaling pathway on proliferation and apoptosis of immature porcine Sertoli cells, SB202190 (SB) was used to induce the inhibition of p38 MAPK signaling pathway. The result from cell cycle analysis showed that SB decreased the cell population in the G1 phase (*p* < 0.05), and enhanced that in the G2 phase (*p* < 0.01) (Figure 9B). Additionally, SB-induced p38 MAPK signaling pathway inhibition significantly upregulated the relative mRNA expression of cell cycle and proliferation-related genes (*p* < 0.05) (Figure 9C,D). Correspondingly, after being treated with SB, cell proliferation activity and relative ATP level were both significantly increased (*p* < 0.01) (Figure 9E,F). Furthermore, a lower cell apoptosis rate was detected in SB-treated cells (*p* < 0.05) (Figure 9G,H). Similarly, the Bcl2 protein expression decreased, whereas the protein expression of BAX and Caspase-3 was enhanced in cells with SB (Figure 9I). These results indicated that miR-24-3p activated the p38 MAPK signaling pathway, whereas SB-induced p38 MAPK signaling pathway inhibition promoted proliferation and inhibited apoptosis in porcine immature Sertoli cells, which was consistent with that of circBTBD7 overexpression. 

## 3. Discussion

**Figure 10:**
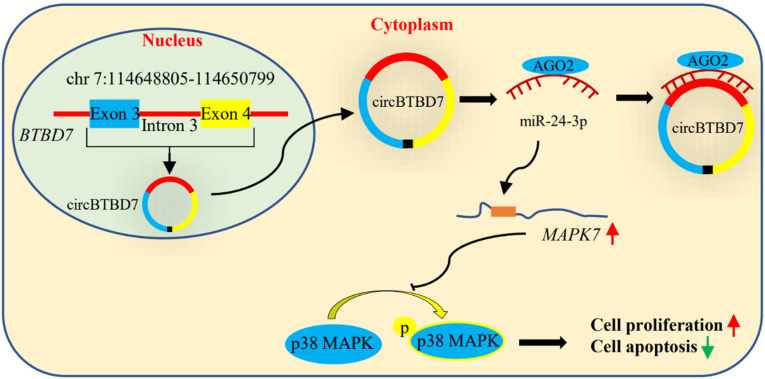
**Model of the main finding in the present study.** circBTBD7 promoted proliferation and suppressed apoptosis in immature porcine Sertoli cells through modulating miR-24-3p/*MAPK7* axis to inactivate p38 MAPK signaling pathway.

Sertoli cells are the key coordinators to guarantee the normal spermatogenesis process, and their number is a major factor in affecting sperm production capacity. Studies have showed that multiple noncoding RNAs are involved in regulating the Sertoli cell proliferation, especially miRNAs [8,10]. Recently, increasing evidence has suggested that circRNA, novel gene regulators, are involved in several biological processes through acting as miRNA sponge [24], including cell proliferation, apoptosis, and differentiation. Based on the development of bioinformatics and high-throughput sequencing strategies, a large proportion of circRNAs have been detected with developmental-stage-specific expression in porcine testicular tissues [16,17,18,25]. However, their functions and mechanisms of action in porcine spermatogenesis remain largely unknown. In the present study, we firstly reported that a novel circRNA named circBTBD7 promoted proliferation and inhibited apoptosis in immature porcine Sertoli cells through modulating miR-24-3p/MAPK7 axis to inactivate p38 MAPK signaling pathway (Figure 10). 

circRNAs have been reported with multiple functions dependent on the locations, parent coding genes, and binding sites, involving sponge for miRNA [24], post-transcriptional regulation [26], peptide or protein biogenesis [27], and splicing interference [28]. However, existing literature has widely reported that the sponge for miRNA is still a major function of circRNAs in regulating cell proliferation, apoptosis, and differentiation. Usually, these circRNAs which act as miRNA sponge are mainly located in the cytoplasm and directly interact with miRNAs [24]. In the present study, the back-spliced junction of circBTBD7 was measured using the RT-PCR and Sanger sequencing strategies. Then, we illustrated that circBTBD7 overexpression promoted cell cycle progression, elevated cell proliferation, and inhibited cell apoptosis in immature porcine Sertoli cells. In addition, circBTBD7 is predominantly located in the cytoplasm and is directly sponged to miR-24-3p. These abovementioned clues indicate that circBTBD7 acted as a sponge of miR-24-3p to participate in regulating immature porcine Sertoli cell growth.

miR-24-3p, highly conserved in animal species, has been witnessed to control the cell proliferation in multiple cell types, including skeletal muscle-derived progenitor cells [29], vascular smooth muscle cells [30], colon cancer cells [31], and human arterial smooth muscle cells [32]. RNA-seq data investigation shows that miR-24-3p exhibits a developmental-stage-specific expression profile in porcine testicular tissues [33,34]. In addition, miR-24-3p is also cloned from the mice Sertoli cells [35], and it also shows a higher expression in porcine Sertoli cells than in other spermatogenic cell types [36]. However, there is no evidence to explore the regulatory role of miR-24-3p in porcine Sertoli cell proliferation. In this study, we demonstrated that miR-24-3p overexpression impeded proliferation and promoted apoptosis of immature porcine Sertoli cells, whereas miR-24-3p inhibition resulted in an opposite effect, which was consistent with circBTBD7 overexpression. Typically, miRNAs induce a post-transcriptional inhibition through targeting the 3′-UTR of protein coding genes. Therefore, to investigate the mechanism of miR-24-3p, its potential target genes were predicted using the TargetScan, miRDB, and miRTarBase online software. Then, the *MAPK7* gene was chosen as a potential target of miR-24-3p. Then, we detected that miR-24-3p directly bound the *MAPK7* gene, and further inhibited the *MAPK7* mRNA abundance, whereas circBTBD7 elevated the MAPK7 protein expression. *MAPK7*, an element of MAPK signaling pathway, is also a supervisor in cell proliferation through activating the transcription factors and modulating the expression of other key genes in the MAPK signaling pathway [37,38]. Our results showed that siRNA-induced *MAPK7* inhibition caused similar effects with miR-24-3p overexpression, and it even attenuated the effects of miR-24-3p inhibition through a co-transfection experiment. Taken together, these clues indicated that miR-24-3p suppressed immature Sertoli cell growth though targeting the *MAPK7* gene, whereas circBTBD7 sponged the miR-24-3p and further upregulated the MAPK7 protein expression.

MAPKs, the members of the Ser/Thr kinase family, are signal transducers to regulate cell proliferation. There are three subfamilies of MAPKs, including JNK, ERK, and p38 MAPK [39]. It has been demonstrated that several key genes are expressed in the Sertoli cells, such as p38 MAPK, ERK1/2, and JNK1/2 [40]. Furthermore, an RNA-seq data investigation showed that these upregulated differential expressed genes of rat immature Sertoli cells enriched in the MAPK signaling pathway, compared with the mature Sertoli cells [41]. These clues indicate that the MAPK signaling pathway participated in the biological processes of Sertoli cells. In the present study, we explored that both miR-24-3p overexpression and *MAPK7* inhibition increases the p-p38 protein expression but had no effect on the total p38 protein expression. It has been widely reported that p38 MAPK is involved in regulating the biological processes of Sertoli cells. For instance, multiple pathogenic factors caused blood–testis barrier disorder through activating p38 MAPK signaling pathway, whereas SB-induced p38 MAPK inhibition abolished their effects [42,43,44]. In this study, the immature Sertoli cells were treated with SB to investigate the regulatory role of p38 MAPK signaling pathway on cell proliferation. The results indicated that inhibition of p38 MAPK signaling pathway promoted the proliferation and inhibited the apoptosis in immature porcine Sertoli cells. These effects were opposite to that of miR-24-3p overexpression and *MAPK7* knockdown, whereas they were consistent with that of circBTBD7 overexpression. 

## 4. Materials and Methods

### 4.1. Cell Culture and Transfection

The immature porcine Sertoli cells were cultured in Dulbecco’s modified Eagle medium (HyClone, Logan, UT, USA) with 10% of fetal bovine serum (Gibco, Grand Island, NY, USA) at 37 °C with 5% of CO_2_. The transfection efficiency of immature porcine Sertoli cells was measured through transfecting riboTRACER™ Fluorescent Oligo and detected using a GE Healthcare IN Cell Analyzer, which reached 60–70% [13]. A total of nine treatments were conducted in the cell transfection, including circBTBD7 vector, vector NC, miR-24-3p mimic, mimic NC, miR-24-3p inhibitor, inhibitor NC, MAPK7 siRNA, siRNA (RiboBio, Guangzhou, China), and SB202190 (SB) (Cell Signaling Technology Corp., Beverly, MA, USA). The circBTBD7 sequence (1994 nt, chr:114648805-114650799, *Sus scrofa* 11.1) was cloned in the pLC5-ciR vector (RiboBio, Guangzhou, China) to conduct the circBTBD7 overexpression vector. A total of 100 pmol (final concentration, 50 nM in the cells) of each abovementioned reagent was diluted with 250 μL of serum-free Opti-MEM (Thermo Fisher Scientific Inc., Grand Island, NY, USA), and incubated at 37 °C for 5 min, respectively. Then, 5 μL Lipofectamine^TM^ 2000 (Invitrogen, Carlsbad, CA, USA) was also diluted with 250 μL serum-free Opti-MEM, and incubated at 37 °C for 5 min. These abovementioned two mixtures were mixed together and incubated at 37 °C for 15 min. Finally, the mixtures were added in each well when the cells reached approximately an 80% confluence. After cultivation for 4 to 6 h at 37 °C with 5% CO_2_, complete medium was used for cultivation. 

### 4.2. Reverse Transcription PCR

Total RNA and genomic DNA of immature Sertoli cells were isolated using TRIzol^®^ reagent (Invitrogen, Carlsbad, CA, USA) and a genomic DNA isolation kit (Beyotime, Shanghai, China). The premiers of circBTBD7 and *GAPDH* genes were designed using Oligo 7.0 (Table S1, see Supplementary Materials) and were synthesized by Sangon Bio. (Shangshai, China). Total RNA was incubated with RNase R (5 U/μg) (Epicentre, Madison, WI, USA) for 15 min at 37 °C to remove linear RNA. The cDNA was synthesized using a PrimeScript first strand cDNA synthesis kit (TaKaRa, Dalian, China) according to the manufacturer’s protocols. Then, the PCR reaction was performed in 20 μL and the 8 μL PCR product separated by 1.5% agarose gel electrophoresis. The PCR product was then extracted using a DNA gel extraction kit (Beyotime, Shanghai, China) and identified using the Sanger sequencing assay (Sangon Bio., Shangshai, China). 

### 4.3. Fluorescence in Situ Hybridization

A FISH probe targeting the back-splicing junction of circBTBD7 was designed to measure its subcellular location (RiboBio, Guangzhou, China). The 4′,6-Diamidino-2-phenylindole (DAPI) was used to mark the nuclei. Immature porcine Sertoli cells were seeded in 96-well culture plate for 24 h, and then hybridization was performed with the FISH probe using a fluorescent in situ hybridization kit (RiboBio, Guangzhou, China) following the manufacturer’s protocols. After washing three times with PBS, the nuclei were marked by staining with DAPI (50 ng/mL) for 5 minutes. The images were captured using a fluorescence microscope (Leica, Weztlar, Germany).

### 4.4. Cell Cycle Assay

After 24 h transfection, cells were washed three times using PBS and harvested in a 1.5 mL centrifuge tube. Then, cells were incubated in 70% (*v/v*) ethanol overnight at −20 °C, and then in propidium iodide (PI) solution (50 mg/mL) for 30 min at 4 °C. The cell suspension was analyzed using a cell cycle testing kit (KeyGen Biotech, Nanjing, China) on a FACSCanto II Flow Cytometer (Becton Dickinson, Trenton, NJ, USA) according to the manufacturer’s protocols.

### 4.5. Cell Proliferation Assay

Cell proliferation was examined using the cell counting kit-8 (CCK-8) (Multiscience, Hangzhou, China) and 5-ethynyl-2′-deoxyuridine (EdU) (RiboBio, Guangzhou, China) incorporation assays. Cells were seeded in a 96-well culture plate at a density of 1 × 10^4^ cells/well in 100 μL culture medium. For the CCK-8 assay, 10 μL CCK-8 medium was added in each well at 0 h or 24 h after transfection. Then, cells were incubated for 4 h at 37 °C. The absorbance value of each well was detected using an ELISA plate reader (Molecular Devices, San Francisco, CA, USA) at 450 nm. For the EdU assay, 100 μL EdU medium (50 μmol) was added in each well 24 h after transfection, and cells were incubated for 2 h at 37 °C. Then, DNA staining solution and EdU staining solution were added in each well to mark living cells (blue) and the proliferating (red) cells according to the manufacturer’s protocols, respectively. We used a fluorescence microscope to observe the cells at 20× and the ImageJ software to determine cell numbers.

### 4.6. Cell Apoptosis Assay

Cell apoptosis was detected using an Annexin V-FITC apoptosis detection kit (KeyGen, Nanjing, Biotech, China) and an adenosine triphosphate (ATP) assay kit (Beyotime, Shanghai, China). Cells were cultured in 6-well plates with 2 mL medium. After 24 h transfection, cells were collected in a 1.5 mL centrifuge tube. Before Annexin V-FITC apoptosis analysis, cells were washed three times and double stained with FITC-Annexin V and PI. Then, cell samples were analyzed using a FACSCanto II Flow Cytometer (Becton Dickinson, Trenton, NJ, USA). Percentages of early apoptosis and late apoptosis cells were counted and used as the cell apoptosis rate. The ATP concentration was evaluated using an ATP assay kit (Beyotime, Shanghai, China) according to the manufacturer’s protocols. The relative ATP level of experimental groups was normalized with the NC group.

### 4.7. RNA Pull Down Assay

Biotin-labeled circBTBD7 and miR-24-3p probes were provided by RiboBio (Guangzhou, China). Then, 4 × 10^7^ circBTBD7 or miR-24-3p overexpressed cells were lysed and incubated with the specific probe or probe NC at 37 °C for 90 min. Then, these samples were incubated with streptavidin magnetic beads (100 μL beads/400 pmol probes) at 26 °C for 30 min. The washed streptavidin magnetic beads were eluted using the RNA-Elution buffer. Finally, the expression of circBTBD7 or miR-24-3p was measured using the qRT-PCR in the immunoprecipitates. 

### 4.8. Real-Time qPCR

Total RNA was extracted using TRIzol reagent (Invitrogen, Carlsbad, CA, USA) according to the manufacturer’s protocols. The primers were designed using Oligo 7.0 software (Table S1) and synthesized by Sango Bio. (Shanghai, China). The cDNA of each sample was synthesized using a Primescript first strand cDNA synthesis kit (TaKaRa, Dalian, China) according to the manufacturer’s protocols. The qRT-PCR amplifications were performed on a Thermo Scientific PIKO REAL 96 real-time PCR System using a SYBR Green kit (TaKaRa, Dalian, China). *pig-TBP* genes were used as internal controls. The relative expression of each gene was evaluated using the 2^−^^△△Ct^ method.

### 4.9. Western Blotting

Total protein was extracted using the radio immunoprecipitation assay (RIPA) lysis buffer (Beyotime, Shanghai, China). Then, the protein concentration was measured using a bicinchoninic acid protein assay kit (BCA) (Beyotime, Shanghai, China) according to the manufacturer’s protocols. The boiled protein samples were electrophoresed on 10% SDS-polyacrylamide gels and then transferred onto a PVDF membrane (Beyotime, Shanghai, China). The membrane containing protein fractions was blocked with 5% nonfat milk for 2 h and then incubated with primary antibodies overnight at 4 °C, including MAPK7 (1:2000, Abcam, Cambridge, MA, USA), Bcl2 (1:1000, Proteintech Group, Chicago, MI, USA), BAX (1:2000, Proteintech Group, Chicago, MI, USA), Caspase-3 (1:100, Abcam, Cambridge, MA, USA), p-p38 (1:500, Proteintech Group, Chicago, MI, USA), p38 (1:500, Abcam, Cambridge, MA, USA), and β-actin (1:2000, Proteintech Group, Chicago, MI, USA). After washing, the membrane was incubated with secondary antibodies (1:5000, Proteintech Group, Chicago, MI, USA) for 2 h at 26 °C. Protein bands were visualized using an ECL advanced Western blotting detection kit (Beyotime, Shanghai, China). β-actin served as the loading control.

### 4.10. Dual-Luciferase Activity Assay

The target site between circBTBD7 and miR-24-3p was predicted using TargetScan online software. A fragment of the circBTBD7 harboring putative binding site of miR-24-3p was amplified using specific primers. Then, a mutant circBTBD7 with a six-base mutation at the putative binding site was also amplified. The abovementioned two fragments were subcloned into pmirGLO dual-luciferase vectors (Promega, Madison, WI, USA). The conducted vectors were co-transfected with miR-24-3p mimic or mimic NC into HEK293T cells. After 48 h transfection, the luciferase activity of each cell group was measured using the Dual-Glo luciferase assay system (Promega, Madison, WI, USA). 

### 4.11. Statistical Analysis

Data are presented as the mean ± standard deviation (SD). Data from experiments with multiple treatment groups were subjected to a one-way ANOVA followed by Duncan’s multiple comparison test of significance using SPSS 17.0 software (IBM, Armonk, NY, USA). The t-test was used to test the differences in experiment with only two treatment groups. *p* < 0.05 or *p* < 0.01was considered statistically significant.

## 5. Conclusions

This study illustrated that circBTBD7, a novel stable circular RNA, promoted proliferation and suppressed apoptosis in immature porcine Sertoli cells through modulating miR-24-3p/*MAPK7* axis to inactivate p38 MAPK signaling pathway. For domestic animals, this study expanded our understanding of noncoding RNAs related to porcine spermatogenesis. From a medical perspective, these cell proliferation-related molecules investigated in the present study have the potential to be therapeutic targets for Sertoli cell disorder-induced male infertility. For instance, aberrant Sertoli cell number and function induced spermatogonia stem cell self-renewal and differentiation disorder, and the pathological changes and maturation disorders of Sertoli cell induced nonobstructive azoospermia. 

## Data Availability

The data analyzed during the current study are available from the corresponding author on reasonable request.

## Supplementary Materials

The following are available online at https://www.mdpi.com/article/10.3390/ijms22179385/s1.

## Abbreviations

circRNA, circular RNA; miRNA, microRNA; 3′-UTR, 3′-untranslated region; NC, negative control; EdU, 5-ethynyl-2’-deoxyuridine; CCK-8, cell counting kit-8; PI, propidium iodide; ATP, adenosine triphosphate; qRT-PCR, real-time quantitative PCR; RT-PCR, reverse transcription PCR; FISH, fluorescence in situ hybridization; PBS, phosphate buffer saline; PI, propidium iodide; siRNA, small interfering RNA; RIPA, radio immunoprecipitation assay; SB, SB202190; DAPI, 4′,6-Diamidino-2-phenylindole; BCA, bicinchoninic acid; SD, standard deviation; *MAPK7*, mitogen-activated protein kinase 7; *BTBD7*, BTB (POZ) domain containing 7; *Bcl2*, B cell leukemia/lymphoma 2; *BAX*, BCL2-associated X; *CDK4*, cyclin dependent kinase 4; *CCND1*, cyclin D1; *IGF*, insulin-like growth factor; *GNDF*, glial cell line derived neurotrophic factor; *PCNA*, proliferating cell nuclear antigen; *EGF*, epidermal growth factor; *c-MYC*, transcriptional regulator Myc-like; *CCNE1*, cyclin E1; *BMP*, bone morphogenetic protein; *FGF*, fibroblast growth factor.
