# Supplementary material for: circBTBD7 Promotes Immature Porcine Sertoli Cell Growth through Modulating miR-24-3p/MAPK7 Axis to Inactivate p38 MAPK Signaling Pathway
Source: Int J Mol Sci. 2021 Aug 30;22(17):9385. doi: 10.3390/ijms22179385 (PMC8431111; doi:10.3390/ijms22179385)
Supplement: Supplementary file 1 [file ijms-22-09385-s001.zip › ijms-1322669-supplementary.pdf]

Table S1 The primer sequences used in the present study

Gene	Primer	Sequence（5`→3`）
ssc-circ-BTBD7	R	ACCCTGGATCCAGTAGCCTCT
	F	GCCAAACATGACCAGGGCAG
ssc-*GAPDH*	R	AAGTCAGGAGATGCTCGGTGT
	F	ATTTGGCTACAGCAACAGGGT
ssc-miR-222	RT	GTCGTATCCAGTGCAGGGTCCGAGGTATTCGCACTG GATACGACGAGACC
	R	AACGCTTCACGAATTTGCGT
	F	ATCGGAGCTACATCTGGCTACTG
ssc-miR-24-3p	RT	GTCGTATCCAGTGCAGGGTCCGAGGTATTCGCACTG GATACGACCTGTTC
	R	AGTGCAGGGTCCGAGGTATT
	F	GCGTGGCTCAGTTCAGCAG
ssc-miR-744	RT	GTCGTATCCAGTGCAGGGTCCGAGGTATTCGCACTG GATACGACTGCTGT
	F	GTGCGGGGCTAGGGCTA
ssc-miR-425-3p	RT	GTCGTATCCAGTGCAGGGTCCGAGGTATTCGCACTG GATACGACGGGCGG
	F	GCGATCGGGAATGTCGTGT
ssc-miR-34a	RT	GTCGTATCCAGTGCAGGGTCCGAGGTATTCGCACTG GATACGACACAACC
	F	CGCGTGGCAGTGTCTTAGCT
ssc-miR-30c	RT	GTCGTATCCAGTGCAGGGTCCGAGGTATTCGCACTG GATACGACAGAGTA
	F	CGCTGGGAGAAGGCTGTT
ssc-miR-219a	RT	GTCGTATCCAGTGCAGGGTCCGAGGTATTCGCACTG GATACGACCGGGAC
	F	GCGCGAGAGTTGAGTCTGGAC
ssc-miR-143-5p	RT	GTCGTATCCAGTGCAGGGTCCGAGGTATTCGCACTG GATACGACCCAGAG
	F	CGGGTGCAGTGCTGCAT
ssc-miR-139-3p	RT	GTCGTATCCAGTGCAGGGTCCGAGGTATTCGCACTG GATACGACACTCCA
	F	GGAGACGCGGCCCTGT
ssc-miR-345	RT	GTCGTATCCAGTGCAGGGTCCGAGGTATTCGCACTG GATACGACCTCCAG
	F	GCGCCCTGAACTAGGGGT
ssc-miR-139-5p	RT	GTCGTATCCAGTGCAGGGTCCGAGGTATTCGCACTG GATACGACCTGGAG
	F	CGCGTCTACAGTGCACGTGT
MAPK7	R	TGGATGACCTGAGCCGAGTGC

Gene	Primer	Sequence（5`→3`）
	F	GGACCTGATGGAGAGCGACCTG
BMP4	R	TGATTCAGCGGCAACCACATCC
	F	GGAGGATGAAGGAGAGAGCTGA
IGF	R	AGGCCAGCTACCGGTTACAT
	F	GAGACCGCTGTGTATCGCATTCC
GDNF	R	GCCTTCTTCCTCTTCCTCCTCCTC
	F	GCGAGCGATGTCAGCACAGAG
EGF	R	AGGAGCAGCAGCAGGACCAG
	F	TACACCGACAACTCCATCCG
CCND1	R	GCCGCCAGGTTCCACTT
	F	CCTGCTGAAGATGCCCATAAC
CCNE1	R	TGCTCTGCTTCTTACTGCTCG
	F	GTGGCCCTCAAGAGCGTAAG
CDK4	R	CAGACATCCATCAGCCGGAC
	F	ATTTGGCCATGGGCGTGAAC
PCNA	R	CTAGTGCCAAGGTGTCTGCAT
	F	ACCAGGTCACTGAGATCCATCCAC
FGF2	R	TTCGGCAACAGCACACCAATCC
	F	AACCCTTGGCTCTCCACGAG
c-MYC	R	ATTCCGACCTTTTGGCAGGG
	F	CCAGGTCATCACCATCGG
β-actin	R	ACATCTGCTGGAAGGTGGAC
	F	AAGTACTCCGTGTGGATCGG
	RT	AACGCTTCACGAATTTGCGT
U6	R	AACGCTTCACGAATTTGCGT
	F	CTCGCTTCGGCAGCACA
